# Efficacy of scrambler therapy in chemotherapy-induced peripheral neuropathy: a systematic review of randomized controlled trials

**DOI:** 10.1186/s43046-026-00347-w

**Published:** 2026-03-02

**Authors:** Adnan Aldawahreh, Mohammad Etoom, Jafar Alshraideh, Muna Salahat

**Affiliations:** 1 School of Nursing, University of Jordan, Amman, Jordan; 2 Department of Applied Health Sciences, Al-Balqa Applied University, Salt, Jordan; 3 Department of Respiratory and Critical Care, Jadara University, Irbid, Jordan

**Keywords:** Calmare therapy, Chemotherapy induced peripheral neuropathy, Scrambler therapy, CIPN, Systematic review, Neuromodulation

## Abstract

**Background:**

Chemotherapy-induced peripheral neuropathy (CIPN) treatment is challenging due to limited and often unsatisfactory therapy options. Scrambler therapy (ST) is gaining interest as treatment option with promising initial results. However, further evaluation is needed to confirm its effectiveness.

**Purpose:**

The purpose of this review was to evaluate the efficacy of scrambler therapy in the management of chemotherapy-induced peripheral neuropathy.

**Methods:**

This systematic review followed the PRISMA guidelines and the Joanna Briggs Institute (JBI) methodology; however, the protocol was not prospectively registered in the PROSPERO database. Electronic databases (CINAHL, Scopus, Web of Science, ScienceDirect, and PubMed) were searched from January 2014 to August 2024. Only English-language randomized controlled trials were eligible. Two reviewers independently screened studies, extracted data, and appraised methodological quality using JBI critical appraisal tools.

**Results:**

Two independent randomized controlled trials, reported across three publications, met the inclusion criteria. The sample size ranged between 35 and 50 participants, and the mean age ranged between 59 and 61 years. The overall quality assessment ranged between low to moderate risk of bias. The reviewed studies demonstrated varying results regarding the efficacy of ST in managing CIPN. Two studies suggest that ST may reduce CIPN symptoms and improve quality of life, although these effects did not reach statistical significance (*p* > 0.05). However, one study’s lack of significant benefit highlights treatment variability and limitations.

**Conclusion:**

Scrambler therapy does not yet meet statistical criteria for a successful treatment CIPN. Further RCTs with large samples, blinding, and extended follow-up is needed to validate ST effectiveness.

## Introduction

Chemotherapy-induced peripheral neuropathy (CIPN) is a devastating adverse effect experienced by cancer patients undergoing chemotherapy, often with long-lasting effects [1]. This condition arises from the neurotoxic effects of certain chemotherapeutic drugs, leading to symptoms such as numbness, pain, tingling, and weakness in the hands and feet [2]. Approximately 30 to 60% of patients receiving neurotoxic chemotherapeutic drugs develop CIPN, with symptoms persisting in one-third of cases after six months [3].

The impact of chemotherapy on the nervous system varies across medication classes, depending on the physical and chemical properties of the agent administered in either single or multiple doses [4]. Patients using platinum-based drugs and taxanes have the highest risk of developing CIPN, up to 70–100% and 87%, respectively [5].

CIPN significantly affects patients’ quality of life and may complicate cancer treatment, leading to dose reductions or discontinuation of chemotherapy [6]. Compared to cancer patients without CIPN, those with CIPN experience higher medical costs and a greater likelihood of disabilities [5]. Additionally, CIPN can increase the risk of falls, pain, and sleep disturbances, impacting psychological well-being and causing functional and physical impairments [3].

The management of CIPN remains a clinical challenge as current treatment options are limited and often unsatisfactory [7]. Traditional pharmaceutical treatments, such as anticonvulsants, antidepressants, and topical medications, have shown varying efficacy and are commonly associated with side effects [3, 5]. Therefore, there is a growing interest in exploring alternative and complementary therapies that may provide symptom relief while minimizing adverse effects [2]. For instance, nutritional supplements such as glutamine and omega-3 fatty acids exhibit potential neuroprotective benefits. However, their effectiveness remains uncertain, attributable to the complex neuropathic mechanisms of various antineoplastic agents and potential drug interactions [8]. Complementary therapies, including acupuncture, reflexology, and sensorimotor training, show promise in symptom alleviation by targeting oxidative stress. Additionally, while medicinal plants with antioxidant properties are frequently studied, their impact remains controversial [9].

Scrambler therapy (ST), also known as Calmare therapy, is a non-invasive, non-pharmacological approach to neuropathic pain, including CIPN [10]. It utilizes a device to deliver electrical stimulation to the nerves, aiming to disrupt pain signals and convert them into non-painful sensations [11]. ST offers a noninvasive alternative for patients who prefer not to use medications or have had intolerable side effects from other treatments [12].

This novel device provides electrostimulation through surface electrodes placed around the pain area, delivering 16 different electrical currents that mimic normal nerve action potentials. The therapy aims to replace “pain” signals with “non-pain” information, promoting pain relief [13]. The device operates based on algorithms that adjust for various factors, including frequency, duration, and amplitude. Unlike traditional therapies, ST does not block nociceptive signals but rather reconditions supraspinal pain centers by transmitting synthetic “nonpain” signals via C-fibers. This process is hypothesized to modulate aberrant pain signaling by delivering non-nociceptive electrical stimuli, thereby influencing pain perception rather than directly altering neuroplastic mechanisms [14]. The therapy is Food and Drug Administration (FDA) cleared and safe, with low electrical charges, a current range of 3.50–5.50 mA, and a voltage range of 6.5–12.5 V [13].

Initial research suggests that ST could offer significant benefits to patients with CIPN, including pain relief, improved function, and enhanced quality of life [10, 15]. However, limited studies have been conducted to assess the efficacy of ST in managing CIPN. Despite promising initial findings, further rigorous evaluation is necessary to confirm the overall effectiveness and reliability of ST for CIPN treatment [12, 13]. This systematic review aims to assess the existing evidence on the efficacy of scrambler therapy for chemotherapy-induced peripheral neuropathy. By synthesizing data from recent high-quality randomized controlled trials, this review aims to evaluate the therapeutic potential of ST, identify research gaps, and provide updated evidence-based recommendations for the use of ST in managing CIPN.

### Review question

What is the efficacy of scrambler therapy compared to standard care in managing chemotherapy-induced peripheral neuropathy symptoms and improving quality of life in adult cancer patients with CIPN?

## Methodology

A search for studies was conducted across six different databases, specifically targeting studies discussing the use of ST in managing CIPN. Two reviewers independently screened and extracted data. The review adheres to the Preferred Reporting Items for Systematic Review and Meta-Analyses (PRISMA) guidelines for experimental studies [16]. To ensure the study’s rigor and quality, the Joanna Briggs Institute (JBI) methodology for conducting systematic reviews, assessing quality, and synthesizing evidence was employed [17].

### Search strategy

The search focused on randomized controlled trials published in peer-reviewed journals relevant to the study keywords from 2014 to 2024. Electronic databases including CINAHL, SCOPUS, Web of Science, Science Direct, PubMed, and Google Scholar were utilized between January 2014 and August 2024. Clinical trial registries were not searched, as the review focused on completed randomized controlled trials with published outcome data in peer-reviewed journals. The PICO (Population, Intervention, Comparative, and Outcomes) framework was used to formulate clinical queries and guide the literature search as shown in Table 1.

**Table 1:** PICO’s statement for the systematic literature search

Population	Adult cancer patients suffering from chemotherapy-induced peripheral neuropathy
Intervention	Scrambler Therapy
Comparison	Standard care for management of chemotherapy-induced peripheral neuropathy
Outcome	Chemotherapy induced peripheral neuropathy symptoms (pain, tingling, and numbness)
	Quality of life

Through the initial search on various electronic databases, relevant keywords were identified. Various keywords like “Scrambler Therapy”, “Calmare Therapy”, and “Chemotherapy induced peripheral neuropathy” were included in the search. To refine the search results, Boolean operators were applied as follows: (“scrambler therapy” OR “Calmare therapy”) AND (“chemotherapy-induced peripheral neuropathy” OR “CIPN”). These operators were used individually and in combination to expand or restrict search results as appropriate.

Moreover, relevant articles were manually searched for in the reference sections, although none were eligible for the review. M.S. and M.E., the reviewers, independently screened the abstracts and titles of the articles for all retrieved studies. Any studies with relevant abstracts and titles were independently reviewed at the full-text level by M.S. and M.E. Any disagreements between the reviewers were resolved by discussing and involving a third reviewer, A.A.

In this current review, the authors employed the Preferred Reporting Items for Meta-Analysis (PRISMA) checklist and flow chart. The authors compared articles based on several criteria, including the availability of abstracts, English language, quantitative research methods, peer-reviewed status, and publication date. Initially, a search using separate keywords yielded 164 articles. After identifying and excluding 105 duplicate articles from the initial pool of 164, a total of 59 unique articles remained.

A thorough evaluation of the titles, abstracts, and keywords was then performed to ensure the inclusion of studies that directly addressed the research question of the review. The primary objective of this evaluation was to identify studies focused on the use of ST for managing CIPN. Additionally, the studies were required to report on CIPN symptoms—specifically pain, numbness, and tingling—as well as quality of life as primary outcomes, given their clinical relevance in assessing the effectiveness of interventions for CIPN management. The application of these criteria resulted in the exclusion of 21 studies due to irrelevance or a failure to focus on CIPN management, leaving 38 studies considered relevant to the review.

**Fig. 1:**
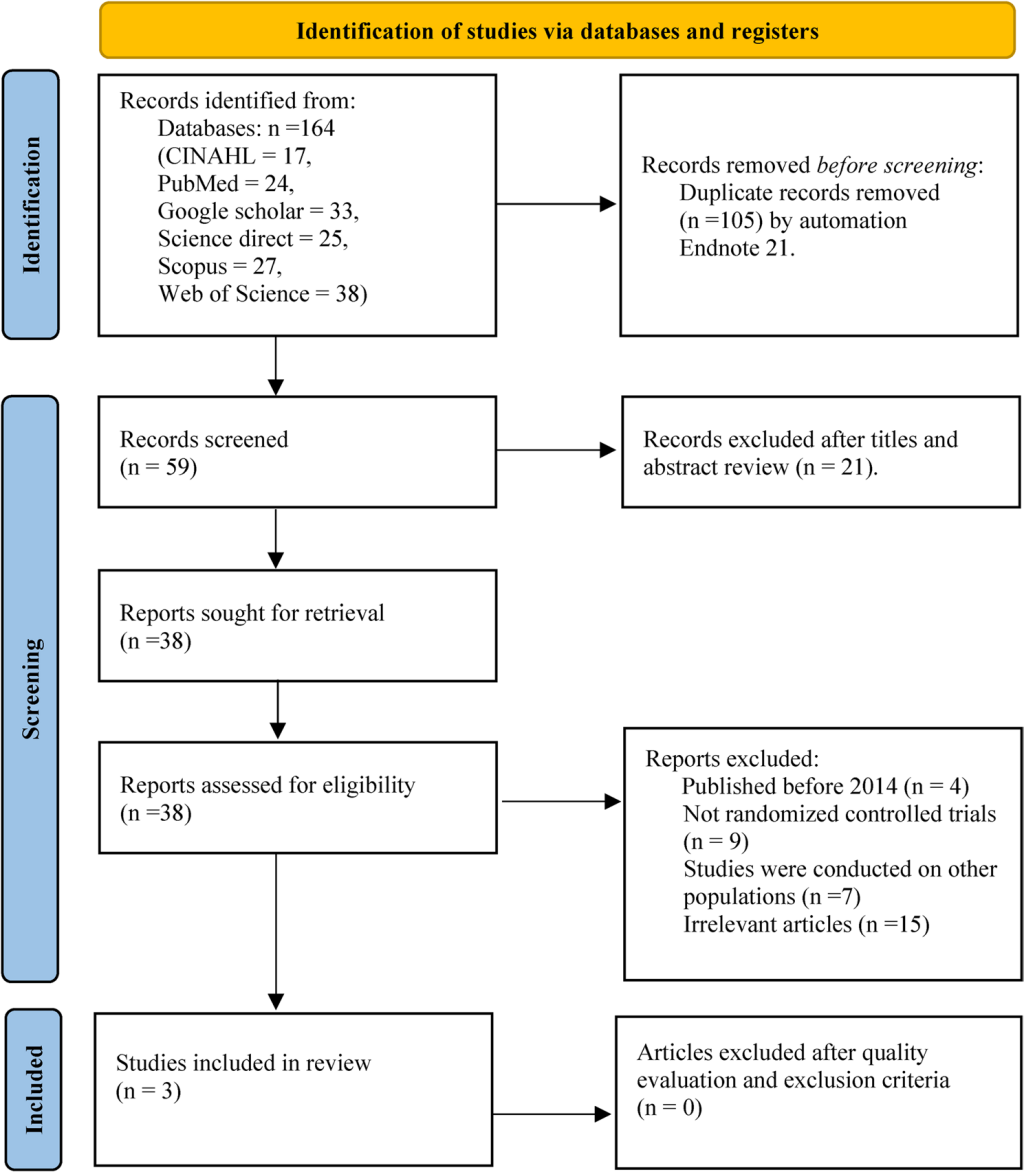
PRISMA checklist

Subsequently, a rigorous evaluation of the full-text articles was conducted to examine study objectives and methodologies, further eliminating articles that did not meet the predefined inclusion and exclusion criteria. These included studies published before 2014, non-randomized designs, the absence of a clear CIPN outcome measure, and studies involving populations that did not meet the eligibility criteria. Ultimately, three randomized controlled trials (RCTs) met all inclusion criteria and were included in the review (Fig. 1).

### Criteria for considering studies for this review

The criteria for inclusion were outlined using the types of studies, participants (P), interventions (I), comparisons (C), and outcomes (O), as shown below:

#### Types of studies

Randomized Controlled Trials (RCTs) published in English between 2014 and 2024.

#### Types of participants

Adults aged ≥ 18 years, diagnosed with CIPN from neurotoxic chemotherapy (including taxanes, platinum-based compounds, vinca alkaloids, or proteasome inhibitors), patients with a stable pain rating of > 4/10 on the pain Numerical Rating Scale (NRS) or bothersome numbness on the CIPN-20 scale, Eastern Cooperative Oncology Group (ECOG) performance status ≤ 2, life expectancy ≥ 3 months, ability to provide informed written consent, must have completed neurotoxic chemotherapy at least 3 months before registration, no further planned neurotoxic chemotherapy for at least 5 months after registration, and pain or symptoms present for > 1 month.

Exclusion criteria were limited to prior treatment with ST, use of an anti-convulsant to prevent seizures, having a history of peripheral neuropathy prior to receiving a chemotherapeutic agent, receiving gabapentin or pregabalin that could not be discontinued before initiation of ST, a sample size less than 10 participants, and failing to clearly report outcome parameters.

#### Types of interventions

Scrambler therapy.

#### Types of comparison measures

Passive comparison (usual care or wait-list without treatment), active comparison (sham or alternative intervention), or no comparison.

#### Types of outcome measures

Chemotherapy induced peripheral neuropathy symptoms (pain, tingling, and numbness) and quality of life.

### Data extraction and synthesis

The data from the eligible studies was independently extracted by two reviewers (A.A. and M.E.). A developed form was used to record the study characteristics and extract the data. This included the study aim, design, sample size, eligibility criteria, comparative groups, results, and level of evidence (Table 2). Information about the randomization and allocation process was also extracted to ensure study validity and minimize biases.

**Table 2:** Main characteristics of the significant results of included studies

Author & Year & Country	Aim	Design	Sample Size	Population		Comparative Groups		Chemotherapy Agent Received	Results	LOE
				Inclusion Criteria	Exclusion Criteria	Intervention Group	Control Group			
Smith et al., (2020) [19]. United States	To determine whether Scrambler Therapy would result in a significant reduction in pain scores compared to sham therapy in patients with CIPN after 28 days	A randomized, sham-controlled Phase II trial	35 patients	Adults aged 18 years or older with cancer. English speakers. ECOG performance status 0–3. Life expectancy greater than 3 months. Documented CIPN neuropathy of greater than 3 months duration. Discontinued the therapy that caused CIPN for at least 3 months. Reported average daily CIPN rating of greater than 4 out of 10.	Patients with implantable drug delivery systems or metal implants such as pacemakers, defibrillators, or cochlear implants. History of myocardial infarction or ischemic heart disease within the past 6 months. History of uncontrolled epilepsy, brain damage, or symptomatic brain metastases. Current use of anticonvulsants (e.g., gabapentin or pregabalin) or recent use without a one-week washout period.	17 patients received Scrambler Therapy	18 patients received sham treatment	Not explicitly mentioned	No significant differences were observed between the Sham and Scrambler Therapy groups at day 10, 28, 60, or 90 for average pain, BPI, or EORTC CIPN-20. Some individual responses were noted during the Scrambler Therapy treatment, but most dissipated by day 30. There was a noted improvement in the sensory subscale of the CIPN-20 at 2 months in the Scrambler Therapy group (*P* = 0.14), though this was not statistically significant.	II
Loprinzi et al., 2020 [12] United States	To evaluate the efficacy of Scrambler Therapy in patients with chronic CIPN of at least moderate severity, and to potentially support a phase III study	A randomized phase II pilot trial	This study accrued 50 patients, 25 to each of the two study arms; 46 patients were evaluable	Adults with CIPN symptoms for at least 3 months. CIPN-related pain or tingling ≥ 4/10 severity in the week prior to registration. Neurotoxic chemotherapy completed at least 3 months prior. Expected tingling or pain ≥ 4/10 at the first treatment. At least 6 months life expectancy. Able to complete questionnaires. Informed written consent. ECOG Performance Status (PS) of 2 or better. Reviewed by study chair or designate as suitable for study treatments.	Pregnant or nursing. Existing operational implantable drug delivery or medical devices. History of myocardial infarction or ischemic heart disease within the past 6 months. History of epilepsy, brain damage, or anticonvulsant use. Skin conditions that prevent proper electrode application. History of peripheral neuropathy prior to neurotoxic chemotherapy. Prior treatment with Scrambler Therapy. Currently receiving gabapentin or pregabalin (must be willing to discontinue).	Scrambler Therapy	Transcutaneous Electrical Nerve Stimulation (TENS)	Paclitaxel, Carboplatin Oxaliplatin, Cisplatin Bortezomib, Docetaxel	40% of the Scrambler Therapy group achieved at least a 50% reduction in pain/tingling scores, compared to 20% in the TENS group (*p* = 0.12). The Scrambler Therapy group showed better improvements in pain, tingling, and numbness scores compared to the TENS group. EORTC QLQ-CIPN20 Sensory Small positive difference favoring Scrambler Therapy (*p* = 0.13). Global Impression of **-** Significant improvements in “neuropathy symptoms” (*p* = 0.001) and pain (*p* = 0.005) during treatment. Patient Scrambler group patients were more likely to recommend their treatment (*p* < 0.0001). Minimal toxicities reported, resolving after treatment.	II
Childs et al., 2021 [5] United States	To evaluate the efficacy of Scrambler therapy versus transcutaneous electrical nerve stimulation in treating chemotherapy-induced peripheral neuropathy	Phase II randomized controlled trial with a two-part crossover design. The trial included a 2-week treatment period followed by an 8-week observation period, and then crossover treatment	50 patients	Age ≥ 18 years. Pain or symptoms of CIPN of ≥ 3 months duration. Neurotoxic chemotherapy must have been completed ≥ 3 months before registration. Tingling or pain of at least 4 out of 10 in severity experienced during the 7 days before registration. Eastern Cooperative Oncology Group Performance Status of 3 or better. Life expectancy greater than 6 months.	Pregnant or nursing. Implantable drug delivery systems, stents, or other metal devices. Seizure disorder or symptomatic brain metastases. Skin conditions or sores that would limit the application of electrodes. Receiving a gabapentinoid that could not be discontinued before protocol therapy initiation. History of neuropathy before receiving chemotherapy. Previously treated with Scrambler therapy.	TENS-treated patients (*n* = 22)	Scrambler-treated patients (*n* = 24)	Paclitaxel, Oxaliplatin, Other/Combination	A 50% or greater reduction in primary symptom (pain or tingling) score was achieved by 60% (6 of 10) of Scrambler-treated patients and 25% (3 of 12) of TENS-treated patients after crossover (*P* = 0.11). By day 4 of treatment, the two arms diverged with respect to mean change in primary symptom score, with this effect largely carried through to the end of the two-week treatment period. Scrambler therapy appeared better than TENS when assessed by Global Impression of Change for neuropathy, pain, and overall quality of life.	II

*LOE * level of evidence, *CIPN * chemotherapy-induced peripheral neuropathy, *ECOG * Eastern Cooperative Oncology Group, *BPI * Brief Pain Inventory, *QST* Quantitative Sensory Testing

This review utilized data synthesis through narrative synthesis. Narrative synthesis entails summarizing the results qualitatively and emphasizing the differences in primary and other outcomes between the control and interventional groups. This method provided a comprehensive overview of the evidence by identifying the variability across studies. The clinical approach to heterogeneity involves assessing the differences in study characteristics, interventions, and populations. The results section transparently presented the heterogeneity to ensure the validity and reliability of the findings [18]. In addition, a meta-analysis was not performed because substantial clinical and methodological heterogeneity was observed among the included randomized controlled trials. The studies differed in comparator groups (sham versus TENS), outcome definitions, timing of pain assessment, analytic approaches, and reporting formats, which precluded the calculation of a consistent and comparable effect size. Furthermore, variations in outcome scales and the absence of sufficiently comparable summary statistics limited the validity of quantitative pooling. Given the small number of trials and the risk of producing potentially misleading pooled estimates, narrative synthesis was considered the most appropriate and methodologically sound approach in accordance with established evidence synthesis guidance.

### Reporting of bias and certainty of findings assessment

The quality of the included studies was assessed using the JBI Critical Appraisal Checklist for RCTs [19]. Two reviewers (M.S., A.A.) independently evaluated the studies’ quality, and a third reviewer (M.E.) stepped in to resolve any disagreements. The studies were categorized as having low, moderate, or high risk of bias based on methodological quality, which involved assessing the randomization process, allocation concealment, blinding of assessors, researchers, and participants, as well as any other potential risk of bias. This classification provides a general assessment without specific cut-off points (Table 3).

**Table 3:** JBI critical appraisal checklist for randomized control trials for included studies

1. True Randomization	[19]	[12]	[5]
	Yes	Yes	Yes
2. Allocation Concealment	Yes	No	No
3. Baseline Similarity	Yes	Yes	Yes
4. Participant Blinding	No	No	No
5. Treatment Delivery Blinding	Unclear	No	No
6. Outcome Assessment Blinding	Yes	No	No
7. Treatment Group Consistency	Yes	No	Unclear
8. Follow-Up Completeness	Yes	Yes	Yes
9. Intention-to-Treat Analysis	Yes	Yes	Unclear
10. Uniform Outcome Measurement	Yes	Yes	Unclear
11. Reliable Outcome Measurement	Yes	Yes	Yes
12. Appropriate Statistical Analysis	Yes	Yes	Yes
13. Trial Design and Deviations	Yes	Yes	Yes
Overall Quality Assessment	Low risk of bias	Moderate risk of bias	Moderate risk of bias

In terms of risk assessment, all three studies utilized true randomization processes, which enhance the validity of the results [12, 15, 20]. Smith et al. [19] had clear allocation concealment, while there was no concealment for either Loprinzi et al. [12] or Childs et al. [5]. All three studies showed baseline similarity among participants.

None of the studies implemented participant blinding. Smith et al. [19] had unclear treatment delivery blinding, while Loprinzi et al. [12] and Childs et al. [5] did not employ this blinding. Outcome assessment blinding was reported only in Smith et al. [19]; in contrast, Loprinzi et al. [12] and Childs et al. [5] were judged at risk of bias because outcomes were self-reported by unblinded participants. Smith et al. [19] demonstrated treatment group consistency, while this was not evident in Loprinzi et al. [12] and it was unclear in Childs et al. [5]. All three studies maintained completeness in their follow-up process. Smith et al. [19] and Loprinzi et al. [12] conducted intention-to-treat analysis, while it was unclear in Childs et al. [5]. Smith et al. [19] and Loprinzi et al. [12] employed uniform outcome measurement, which was unclear in Childs et al. [5]. All three studies utilized reliable outcome measurement. Appropriate statistical analysis was evident in all three studies. All studies were in adherence with the trial design and deviations. To sum up, overall quality assessment exhibited low risk of bias for Smith et al. [19] study, while moderate risk of bias for both Loprinzi et al. [12] and Childs et al. [5].

## Results

### Sample characteristics

The mean patient’s age ranges between 59 and 61 years. The sample size in the selected studies ranges between 35 and 50 participants. In the first study, Smith and his colleagues recruited a total of 35 patients (9 men and 26 women), with 17 patients randomized to ST and 18 to ‘‘sham’’ [20]. The second study included 50 patients (12 men and 34 women), with 25 patients randomized to each of the two study arms [12]. The third study included 50 participants. However, only 22 participants completed the cross-over phase (five men and 17 women), with 12 patients treated with TENS and 10 patients treated with ST [15].

### Main results

Smith et al. [19] conducted a randomized, sham-controlled Phase II trial to determine whether Scrambler Therapy (ST) would result in a significant reduction in pain scores compared to sham therapy in patients with chronic chemotherapy-induced peripheral neuropathy (CIPN) after 28 days. A total of 35 patients were randomized, with 17 assigned to the ST group and 18 to the sham group. The primary endpoint was the change in the average pain score (measured on a 0–10 Numeric Rating Scale) from baseline to day 28. The results revealed no statistically significant difference between the groups for this primary outcome, with a difference in the change scores of -0.56 (90% one-sided confidence interval: -1.42 to Infinity; *p* = 0.80), indicating the sham group had a slightly greater, but not significant, reduction. Furthermore, no significant differences were found at days 10, 60, or 90 for average pain, or for secondary outcomes measured by the Brief Pain Inventory (BPI)-CIPN and the EORTC CIPN-20 scales. The researchers identified several potential reasons for the observed null outcomes, including: the possible ineffectiveness of ST for CIPN; a potential therapeutic effect from the sham procedure itself; the small sample size combined with patient heterogeneity; the suboptimal placement of electrodes on nonpainful but damaged nerves; or a combination of these factors [20].

Loprinzi et al. [12] conducted a randomized phase II pilot trial, to evaluate the efficacy of ST in patients with chronic CIPN of at least moderate severity. This publication represents the primary report of a single randomized trial. This study accrued 50 patients, 25 to each of the two study arms; 46 patients were evaluable. Patients were permitted to crossover to the alternative treatment after the completion of the full 2-week treatment period and the subsequent 8 weeks of observation. However, the authors indicated that the data from this protocol is currently under analysis. The authors utilized NRS, EORTC CIPN-20, and Subject Global Impression of Change, to assess the pain level and CIPN symptoms. The results revealed that the proportion of patients treated with ST who experienced at least a 50% improvement in baseline pain, tingling, and numbness scores during the two-week treatment period was twice as high as that of patients treated with Transcutaneous Electrical Nerve Stimulation (TENS) therapy. Specifically, the improvement rates ranged from 36% to 56% for Scrambler-treated patients, compared to 16% to 28% for TENS-treated patients (*p* = 0.12). Additionally, Global Impression of Change scores for neuropathy symptoms, pain, and quality of life showed similar improvements during the entire study period. Furthermore, patients in the Scrambler group were more likely to recommend their treatment to others, both during the two-week treatment period and the eight-week follow-up period (*p* < 0.0001) [12].

Childs et al. [5] reported a secondary analysis of a phase II randomized controlled trial with a two-part crossover design to evaluate the efficacy of ST versus TENS in treating CIPN. This publication does not represent an independent trial, but rather reports crossover-phase results from the original Loprinzi et al. [12] cohort. After completion of the first phase of the study, patients were allowed to crossover and receive the alternate treatment. The same procedure was followed for an additional 10 weeks (2 weeks of active therapy plus 8 weeks of observation). The total number of participants in the initial phase of the study, as reported by Loprinzi et al. [12], was 50: TENS-treated patients (*n* = 22) and scrambler-treated patients (*n* = 24); four participants didn’t complete the initial phase of treatment. Additionally, during this crossover phase, only 22 participants completed the treatment: TENS-treated patients (*n* = 12) and scrambler-treated patients (*n* = 10). The primary assessment methods utilized were patient-reported outcomes, encompassing symptom severity scales and Global Impression of Change questionnaires. Symptom severity was evaluated daily throughout the treatment period and on a weekly basis during the subsequent eight-week observation period. The primary outcome in the initial phase of the study was defined as a reduction of 50% or greater in primary symptom scores (pain, numbness, and tingling). In this subsequent phase, a reduction was noted in 60% (6 out of 10) of patients treated with ST, compared to 25% (3 out of 12) of patients undergoing TENS therapy following crossover (*p* = 0.11). Treatment results were extremely similar for those who were initially randomized to ST and for those who received it following crossover. By the fourth day of treatment, a divergence in the mean change in primary symptom scores between the two groups was noted, with this trend persisting until the conclusion of the two-week treatment period. ST demonstrated superior outcomes compared to TENS, as evaluated by the Global Impression of Change for neuropathy, pain, and overall quality of life [15].

It is important to note that the studies by Loprinzi et al. [12] and Childs et al. [5] report sequential phases—the initial randomization and the crossover phase, respectively—of a single clinical trial. Consequently, their findings are not derived from independent patient cohorts but represent complementary analyses of the same study population. While this design enhances the internal consistency of the results, as similar outcome patterns were observed across both phases of the same trial, it does not increase the overall strength or quantity of the evidence, which remains primarily based on one moderate-sized phase II study.

## Discussion

By conducting a systematic evaluation of two independent randomized trials (reported in three publications) on the efficacy of ST in CIPN management, we get a thorough view of its effectiveness across several outcomes, reflecting both its potential advantages and limits. The primary outcomes were CIPN symptoms (pain, tingling, and numbness) and overall quality of life.

The implementation of ST was conducted in approximate accordance with the protocol outlined by Pachman et al. (2015) in their single-arm trials. On the initial day of treatment, the most symptomatic area of CIPN was identified based on patient self-reporting. Electrodes, similar to electrocardiogram patches, were placed externally along the lines of pain and/or tingling. Once a pair of electrodes were appropriately positioned, the device was activated, and the intensity was gradually increased to the maximum level tolerable by the patient. In cases where no symptom improvement was observed, the device was deactivated, and the electrodes were repositioned. Upon achieving optimal initial electrode placement, resulting in symptom reduction, additional electrode sets were placed to further encompass the symptomatic area. Up to five channels or sets of electrodes could be utilized during the treatment. Once all channels were used, or a significant reduction in symptoms was achieved, the treatment session continued for 30 min. Each patient underwent daily sessions for up to ten consecutive days, with the option to discontinue treatment if significant benefits were not observed [13].

Loprinzi et al. [12] and Childs et al. [5] found that ST has the potential to reduce CIPN symptoms such as pain, numbness, and tingling while also improving overall quality of life [12, 15]. Loprinzi’s experiment found that ST considerably decreased these symptoms compared to TENS, with a higher proportion of patients seeing a 50% or more reduction in their CIPN symptoms [12]. Similarly, Childs’ study supported these findings by demonstrating that 60% of patients exhibited a reduction in their primary symptom ratings following ST, compared to just 25% in the TENS group, emphasizing ST’s higher efficacy in alleviating CIPN-related symptoms at eight weeks post-treatment [15]. However, their primary endpoints did not reach conventional statistical significance, largely due to being pilot studies with small sample sizes.

In contrast, a study by Smith et al. [19] provided a more cautious stance. Despite ST’s promising premise, Smith’s experiment demonstrated no meaningful difference in pain reduction between ST and sham treatments at various follow-up intervals. This disparity might be attributable to a variety of variables, including a limited sample size, possible placebo effects in the sham group, or methodological difficulties such as electrode positioning. The authors suggest that putting the electrodes close to the painful area but in areas of numbness or altered sensation may have affected the transmission of the “nonpain” signal. This may have diminished the efficacy of ST. Damaged neurons—numbed but not painful—may have failed to transmit impulses. However, this explanation aligned with a previous study that found nerve damage up the leg, so moving the electrodes higher on the L5 and S1 dermatomes improves scrambled nonpain impulse transmission and pain relief [21]. To address this, the authors recommend starting in the L5 dermatome above any altered sensation, usually on the thigh. Smith’s results suggest that while ST may offer some initial relief, its overall effectiveness compared to a placebo remains uncertain, underscoring the need for further investigation to validate its efficacy [20].

Quality of life, as assessed by patient-reported outcomes and the Global Impression of Change, yielded inconsistent results across trials. In the study done by Smith et al. [19], no significant increases in quality-of-life indicators were seen between the sham and true ST groups. However, Loprinzi et al. [12] and Childs et al. [5] studies found that patients in the ST group experienced a higher quality of life than those in the TENS group. The Global Impression of Change ratings showed that patients who received ST experienced improvements in neuropathic symptoms, pain, and overall quality of life [12, 15].

Although RCTs investigating ST for CIPN have concentrated on symptom relief within a three-month period, there is a notable lack of evidence regarding the therapy’s long-term effectiveness. Specifically, it remains uncertain whether symptoms may recur post-treatment, particularly among individuals who experience pain relief during the treatment and observation phases. Therefore, further research is imperative to investigate the long-term effects and the durability of symptom relief provided by ST in CIPN management.

The ST is generally safe neuromodulation method, with only limited reported adverse events limited to minor skin-related issues, such as contact dermatitis and ecchymosis, with no serious complications documented [22]. For instance, only single case of ecchymosis at electrode placement sites was reported by Loprinzi et al. [12], with no severe adverse events observed.

These results highlight the difficulty of assessing the efficacy of ST for CIPN. While evidence from Loprinzi et al.‘s [12] and Childs et al.‘s [5] studies suggests that ST can reduce CIPN symptoms and improve quality of life, the lack of significant benefit observed in Smith et al.‘s [19] study highlights the limitations and potential variability in treatment response.

Despite the application of rigorous selection criteria, we recognize that substantial variations exist among the studies in terms of sample size, data collection methodologies, blinding procedures, and statistical approaches. Notably, two studies exhibited a moderate risk of bias with strong methodological rigor, while one study presented a low risk of bias due to limitations in allocation concealment, intention-to-treat analysis, and outcome assessment blinding. The methodological variability across studies necessitates caution in interpreting the findings. Such inconsistencies could affect the robustness of the conclusions, particularly concerning the treatment’s effectiveness and the generalizability of the results. To address these discrepancies, future research should aim to adopt more standardized and rigorous study designs, incorporating appropriate sample sizes, blinding procedures, and statistical methodologies. These improvements would enhance the reliability of the findings and contribute to a more accurate assessment of ST’s efficacy in CIPN management.

### Implications and limitations

The implications of this review are relevant for both clinical practice and future research. While ST has been explored as a non-pharmacological approach for managing CIPN, the current RCTs evidence does not provide firm statistical support for its effectiveness. Therefore, ST should be regarded as an investigational intervention rather than an established treatment option for CIPN. Clinicians should exercise caution when considering its use and clearly communicate the current uncertainty regarding its benefits.

Several limitations should be acknowledged. Two of the three included publications originate from the same trial, which limits independent replication of the findings. The small sample sizes and methodological heterogeneity across trials limit the robustness and generalizability of the findings. The absence of a meta-analysis reflects substantial clinical and methodological variability, which precluded meaningful quantitative pooling. Outcomes relied largely on subjective patient-reported measures, often assessed without adequate blinding, increasing susceptibility to placebo effects. In addition, relatively short follow-up periods across studies restrict conclusions regarding the durability of treatment effects. Furthermore, although ST was introduced before 2014, the search was limited to 2014–2024 to capture more mature evidence, which may have excluded earlier relevant studies, while the absence of clinical trial registry searches may have led to the omission of unpublished or negative trials, thereby increasing the potential for publication bias. Collectively, these limitations underscore the need for larger, rigorously designed randomized trials with objective outcomes, appropriate blinding, and longer follow-up.

## Conclusion

In conclusion, scrambler therapy does not yet meet statistical criteria for a successful treatment of chemotherapy-induced peripheral neuropathy. Across the two available independent randomized controlled trials (reported in three publications), there is no firm statistical evidence that scrambler therapy is superior to control or sham interventions. Further randomized controlled trials with large sample sizes, appropriate blinding procedures, rigorous methodology, and prolonged follow-up periods are required to validate the effectiveness of scrambler therapy among patients with chemotherapy-induced peripheral neuropathy.

## Data Availability

Not applicable.
